# Quantitative estimation of Nipah virus replication kinetics *in vitro*

**DOI:** 10.1186/1743-422X-3-47

**Published:** 2006-06-19

**Authors:** Li-Yen Chang, AR Mohd Ali, Sharifah Syed Hassan, Sazaly AbuBakar

**Affiliations:** 1 Center for Proteomics Research, Department of Forest Biotechnology, Forest Research Institute, 52109, Selangor, Malaysia; 2 Veterinary Research Institute, Jalan Sultan Azlan Shah, 13800 Ipoh, Perak, Malaysia; 3 Department of Medical Microbiology, Faculty of Medicine, University Malaya, 50603, Kuala Lumpur, Malaysia

## Abstract

**Background:**

Nipah virus is a zoonotic virus isolated from an outbreak in Malaysia in 1998. The virus causes infections in humans, pigs, and several other domestic animals. It has also been isolated from fruit bats. The pathogenesis of Nipah virus infection is still not well described. In the present study, Nipah virus replication kinetics were estimated from infection of African green monkey kidney cells (Vero) using the one-step SYBR^® ^Green I-based quantitative real-time reverse transcriptase-polymerase chain reaction (qRT-PCR) assay.

**Results:**

The qRT-PCR had a dynamic range of at least seven orders of magnitude and can detect Nipah virus from as low as one PFU/μL. Following initiation of infection, it was estimated that Nipah virus RNA doubles at every ~40 minutes and attained peak intracellular virus RNA level of ~8.4 log PFU/μL at about 32 hours post-infection (PI). Significant extracellular Nipah virus RNA release occurred only after 8 hours PI and the level peaked at ~7.9 log PFU/μL at 64 hours PI. The estimated rate of Nipah virus RNA released into the cell culture medium was ~0.07 log PFU/μL per hour and less than 10% of the released Nipah virus RNA was infectious.

**Conclusion:**

The SYBR^® ^Green I-based qRT-PCR assay enabled quantitative assessment of Nipah virus RNA synthesis in Vero cells. A low rate of Nipah virus extracellular RNA release and low infectious virus yield together with extensive syncytial formation during the infection support a cell-to-cell spread mechanism for Nipah virus infection.

## Background

Nipah virus, an enveloped, non-segmented, negative-stranded RNA virus is a recently discovered zoonotic virus belonging to the genus *Henipavirus *of the *Paramyxoviridae *family [1,2]. The virus was initially isolated from an outbreak in Malaysia in 1998 among pig farmers who succumbed to infection characterized by severe encephalitis with high mortality rates [3-5]. No Nipah virus infection was reported since then in Malaysia but sporadic outbreaks of Nipah virus-liked infections were reported in India in 2001 [6] and in Bangladesh in 2001, 2003, and 2004 [7-10]. In the most recent outbreak in Bangladesh more than 40 people were reported ill with Nipah virus-liked encephalitis. Serological tests performed on these patients' samples suggested that they had Nipah virus antibodies [8,9] and Nipah virus isolated from these patients had 91.8% genome sequence similarity to the virus obtained from the outbreak in Malaysia [11]. The origin of Nipah virus is presently unknown. Virus with high sequence similarity to Nipah virus was isolated from flying foxes in Malaysia and Cambodia [12,13] and sero-prevalence studies also revealed the presence of antibodies reactive to Nipah virus amongst these bats in Malaysia, Cambodia and Thailand [13-16]. These suggest the possibility that bats particularly fruit bats could be the natural reservoir for Nipah virus [13,17]. During the Malaysia 1998 outbreak, pigs were identified as the main source of human Nipah virus infections [18,19] and this was supported by the findings that the genome sequence of Nipah virus of pigs and humans were almost identical [20] and culling of all suspected infected pigs effectively eliminated the infection in humans [4]. There were reports of Nipah virus infection in domestic animals including dogs, cats, and horses [4,14,18] and experimental inoculation of pigs and cats [21,22]. The efficiency of virus replication in these animals, however, is not known as methods for detecting the virus are presently limited to qualitative methods; including virus isolation from tissue culture cells, immunohistochemistry, electron microscopy, serum neutralization tests, and ELISA [23]. Application of the polymerase chain reaction (PCR) amplification [24] and fluorogenic real-time reverse transcriptase-PCR (RT-PCR) using Taqman™ [25] for the detection of Nipah virus were only recently described. In the present study, the SYBR^® ^Green I dye-based quantitative real-time RT-PCR (qRT-PCR) amplification assay was established and the assay was used to examine the kinetics of Nipah virus replication in cultured African green monkey kidney (Vero) cells.

## Results

### Nipah virus infection

**Figure 1:**
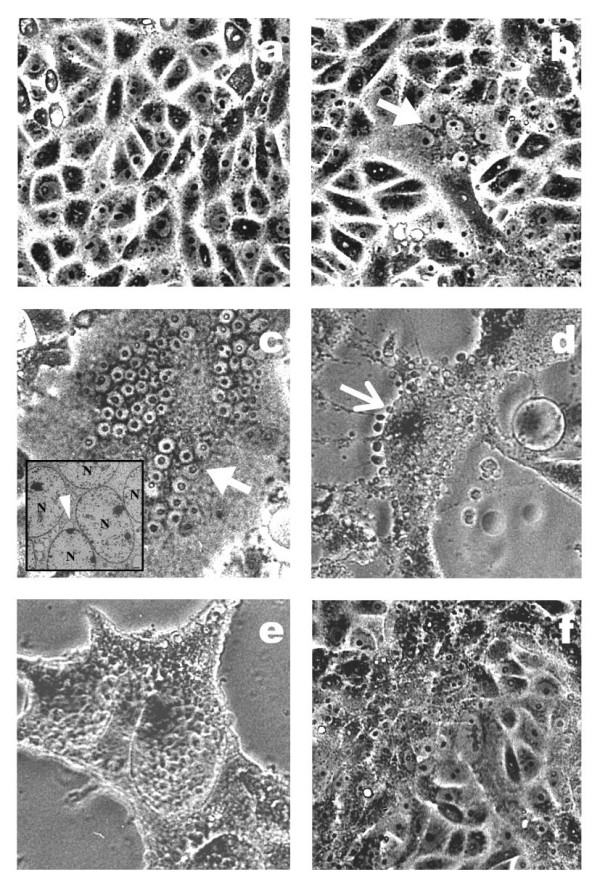
Changes in Vero cell morphology following Nipah virus infection. Cell fusion and syncytial formation were observed at eight hours PI (b, thick arrow). Multinucleated giant cells were noted to increase in frequency at 32 hours PI (c, thick arrow). Evidence of apoptosis with the presence of blebbing cell and apoptotic bodies was noted at 48 hours PI (d, thin arrow). At 64 hours PI onwards, cells started to detach from the surface of the tissue culture flask (e). The inset in (c) is an electron micrograph showing multinucleated cells (N) at 32 hours PI and the presence of nuclear invagination (thin arrowhead). The mock-infected Vero cells at 72 hours PI is shown in (f).

Nipah virus infected Vero cells showed significant cellular morphological changes beginning at eight hours post-infection (PI). Cell fusion and syncytial formation were noted and the frequency of these giant multinucleated cells increased as the infection progressed (Figure 1b, 1c). At 48 hours PI, cells with dendritic-liked projections appeared (Figure 1d) and at 64 hours PI, extensive cell damage occurred and cells were detached from the surface of the tissue culture flask (Figure 1e). There was no obvious cell lysis but evidence of apoptosis such as nuclear invagination (Figure 1c, inset) and membrane blebbing (Figure 1d) were observed.

### RT-PCR for amplification of Nipah virus N gene sequence

**Figure 2:**
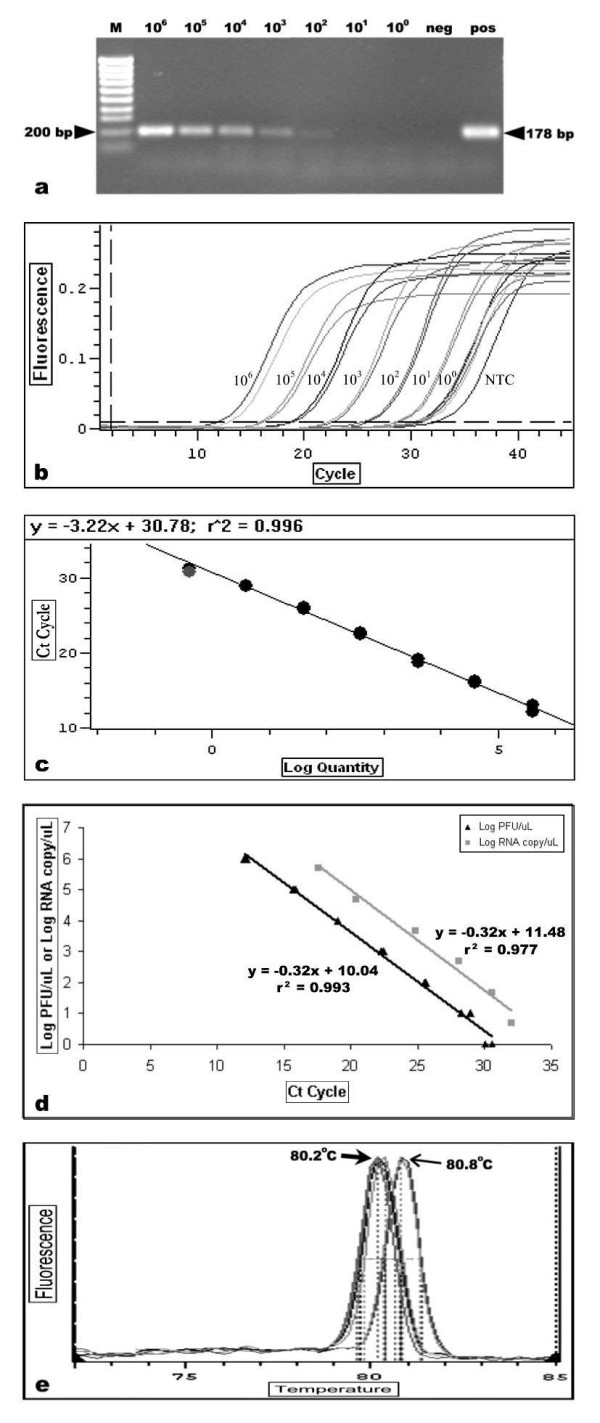
Sensitivity and specificity of one-tube qRT-PCR for detection of Nipah virus RNA. DNA fragments obtained from the RT-PCR were visualized in ethidium bromide-stained agarose gel (a). Input Nipah virus RNA in equivalent log PFU is indicated above the lanes. RNA extracted from mock-infected Vero cells and the Nipah virus Armored RNA^® ^served as the negative (neg) and positive (pos) controls, respectively. Lane (M) consisted of DNA molecular mass marker. Amplification plot of the SYBR^® ^Green I dye-based qRT-PCR assay were obtained from tenfold serial diluted Nipah virus RNA (1 × 10^6 ^to 1 PFU) as indicated in (b). RNA extracted from mock-infected Vero cells was used as the negative control (NTC). The standard curve for the qRT-PCR (c) was generated using the same dilution series of Nipah virus RNA as the amplification plot. Correlation between log PFU/μL of infectious virus against total copy number of Nipah virus RNA (log RNA copy/μL) obtained from the qRT-PCR is shown in (d). Specificity of the assay was assessed and the difference in the melting temperature of the amplified DNA of Nipah virus (thick arrow) and Hendra virus (thin arrow) is indicated in the melting curve analysis (e).

The NIP-NF3 and NIP-NR1 primer set designed for the study amplified the Nipah virus nucleocapsid (N) gene sequence to give a fragment of ~178 bp. The detection limit of this one-tube RT-PCR system was at ~100 PFU/μL and has a dynamic range of five logs (Figure 2a). This was true when it was assessed using a tenfold serially diluted Nipah virus RNA of a virus inoculum with a titer of ~1.0 × 10^7 ^PFU/mL. Nipah virus RNA was detected from as low as one PFU/μL using the similarly diluted RNA in the qRT-PCR assay (Figure 2b). The amplification had a linear detection range of up to 1 × 10^6 ^PFU/μL (Figure 2b). Beyond the detection limit and in the absence of amplification template, non-specific fluorescent signals due to binding of the SYBR^® ^Green I dye to the primer-dimers were observed. This non-specific fragment had a consistent melting temperature (T_m_) value of 76°C and DNA sequencing of the fragment verified its identity (data not shown). The standard curve plot determined using the tenfold serial dilutions of the Nipah virus RNA had a coefficient of correlation of r^2 ^= 0.996 (Figure 2c). In addition, the coefficients of variation (CV) between the different amplifications were low (< 2% and 4% for the intra- and inter-assays, respectively, Table 1). The CV values for the intra-assay, performed in duplicates using the tenfold serially diluted Nipah virus RNA were between 0.01 to 1.67% and the inter-assay variation values collected from 14 independently performed experiments (extraction of RNA and SYBR^® ^Green I-based qRT-PCR amplification assay) were in the range of 1.26 to 3.55%. These suggested a very high reproducibility of the SYBR^® ^Green I-based qRT-PCR amplification assay. Using the commercially available Nipah virus Armored RNA^® ^(Ambion, USA), it was determined that the SYBR^® ^Green I-based qRT-PCR had a sensitivity of five to 5 × 10^5 ^RNA copies/μL in RNA copy number. A linear correlation between the RNA extracted from Nipah virus inoculum (with a virus titer of ~1.0 × 10^7 ^PFU/mL) and the Nipah virus Armored RNA^® ^(RNA copy number) was established (Figure 2d). Using the standard plot, a 1 × 10^6 ^PFU/μL of Nipah virus inoculum corresponded to ~2 × 10^7 ^RNA copies/μL. The higher RNA copy number in the virus inoculum (as opposed to the 1:1 ratio) was expected, as the primers could not differentiate between infectious and noninfectious RNA-containing virus particles such as the defective interfering particle. No amplification, however, was obtained when the genomic RNA of human parainfluenza virus type-3, dengue virus type-2, human enterovirus 71 and Japanese encephalitis virus were used in the SYBR^® ^Green I-based qRT-PCR amplification. On the other hand, fluorescence signals indicating amplification was obtained when Hendra virus genomic RNA template was used. A DNA fragment with a T_m _value of 80.8°C was consistently obtained using the NIP-NF3 and NIP-NR1 primer pairs (Figure 2e). The T_m _value was 0.6°C higher than that obtained from all the amplifications of the Nipah virus RNA. The presence of the Nipah virus and Hendra virus RNA in the respective samples was confirmed by sequencing of the amplified DNA fragments (data not shown).

**Table 1:** Reproducibility of the SYBR^® ^Green I dye-based qRT-PCR assay for the detection and quantification of Nipah virus RNA. Intra- and inter-assay variations were calculated using duplicates and at least 14 replicates, respectively.

CV (%)	PFU/mL							
	
	1 × 10^-1^	1 × 10^0^	1 × 10^1^	1 × 10^2^	1 × 10^3^	1 × 10^4^	1 × 10^5^	1 × 10^6^
Intra-assay	3.27	1.20	1.67	0.06	0.43	0.01	0.79	0.96
Inter-assay	4.76	3.55	2.72	3.52	1.26	1.27	2.11	2.86

### Nipah virus RNA synthesis in Vero cells

**Figure 3:**
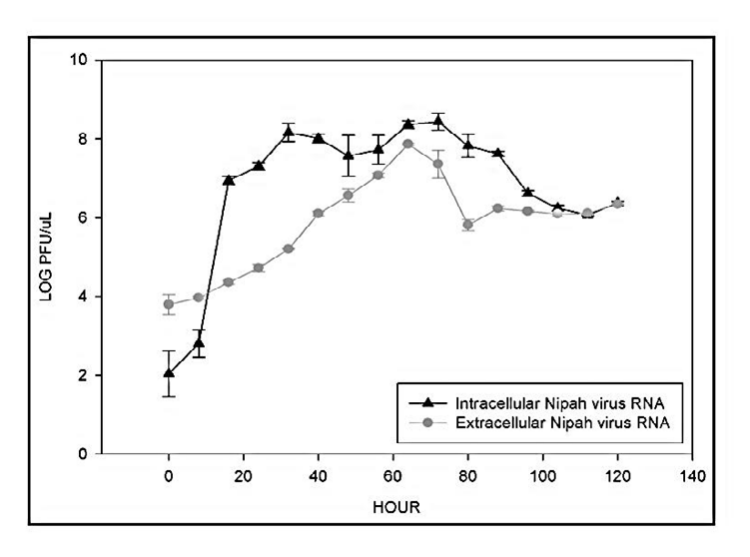
Nipah virus replication in Vero cells. Vero cells were infected with Nipah virus at MOI of 0.2. At selected intervals, total RNA was isolated and the Nipah virus RNA levels were quantified using the SYBR^® ^Green I-based qRT-PCR assay in equivalent log PFU. A latent phase of at least eight hours followed by an exponential increase in the virus RNA level were noted for the intracellular Nipah virus RNA.

The kinetics of Nipah virus RNA synthesis in infected Vero cells was examined quantitatively by determining the amount of intra- and extracellular Nipah virus RNA using the SYBR^® ^Green I-based qRT-PCR amplification. The standard plots for this assay was established using RNA extracted from Nipah virus inoculum with an estimated titer of ~1.0 × 10^7 ^PFU/mL. The amount of viral RNA from the amplification assay was expressed as equivalent log PFU/μL as we were interested only in the proportion of virus RNA that corresponded to the estimated number of infectious virus particles. A significant increase in the intracellular Nipah virus RNA level was noted beginning at eight hours PI (Figure 3). The increase was exponential from 3 log PFU/μL to 7 log PFU/μL or from 1.9 × 10^3 ^to 9.9 × 10^6 ^PFU/μL within the next eight hours. The Nipah virus RNA doubling time during the logarithmic increase was estimated at every ~40 minutes until it reached a steady state at ~7 to 8 log PFU/μL at 32 hrs PI. The RNA doubling time was estimated following the calculations as previously reported [26,27]. The maximum level of RNA at ~8.4 log PFU/μL was detected at 64 hours PI. The intracellular virus RNA level decreased substantially thereafter, as the infection spreads throughout the cell culture flask and this coincided with the extensive formation of large multinucleated syncytial cells. Under our experimental conditions, the extracellular Nipah virus RNA level remained at ~3 log PFU/μL during the first eight hours PI (Figure 3). The extracellular virus RNA level increased progressively at an estimated rate of about 0.07 log PFU/μL per hour to reach a maximum of ~7.9 log PFU/μL at 64 hours PI and this corresponded directly with the time at which the amount of the intracellular virus RNA was at its highest (~8.4 log PFU/μL). A gradual decrease in the level of extracellular virus RNA was observed thereafter and this mirrored the decrease of the intracellular Nipah virus RNA. Using the estimated total extracellular virus RNA copies (obtained from the assay performed using the Nipah virus Armored RNA^®^) and the calculated infectious virus particles in PFU, it was determined that on average, at least 10% (± 0.1%) of the released virus in the cell culture supernatant was infectious.

## Discussion

Findings from the present study reaffirmed earlier reports that Nipah virus replicated productively in Vero cells. In this study, however, the kinetics of the virus replication was estimated using the SYBR^® ^Green I-based qRT-PCR amplification assay. The method was sensitive, highly reproducible and specific enough to differentiate against most other RNA viruses except Hendra virus, a closely related zoonotic virus. The characteristic shift in the T_m _value that differentiates Nipah virus from Hendra virus was obtained perhaps due to the differences in the G+C content of the amplified DNA fragment. The identity of the amplified fragment was confirmed by DNA sequencing. The ability to amplify and differentiate Hendra virus from Nipah virus using the melt curve analysis is potentially useful in the surveillance of Nipah virus infection in animals, particularly in wild animals as both viruses are known to have a common reservoir host, fruit bats [12-16,28]. Furthermore, the primers could also be useful in situations where the N gene sequence of the henipavirus may not be identical to known Nipah and Hendra viruses. For example, the newly reported Nipah virus isolate obtained from human samples in Bangladesh [11] had only 94.3% sequence similarity in its N gene to all the known Nipah viruses. This is in contrast to the earlier report for the quantitative assessment of Nipah virus replication using the TaqMan™ real-time RT-PCR where a highly specific Nipah virus primer pair was used [25]. In that study, the method developed was useful particularly for application in the diagnostic laboratory for the confirmation of Nipah virus infection. The use of this detection method as a routine laboratory test, however, is limited as it is expensive to perform and is associated with potentially high false-negative due to its inability to detect genome nucleotide variations [29].

Results from the quantification of the intra- and extracellular Nipah virus RNA synthesis using the SYBR^® ^Green I-based qRT-PCR suggest that there was a latent phase of at least eight hours following initiation of infection, a period during which no significant increase in Nipah virus RNA synthesis could be detected in the infected Vero cells. A rapid rise in the intracellular virus RNA level occurred within the next eight hours PI and this exponential rise is comparable to that of other virulent paramyxoviruses [30,31]. The short RNA synthesis doubling time during the exponential phase may indicate efficient activities of the newly synthesized viral RNA polymerases [31]. Although the method used in the present study could not differentiate between the virus genomic RNA from the virus mRNAs and the replicative forms, it is nonetheless a reflection of increased Nipah virus RNA synthesis in the infected Vero cells and these results are comparable to that previously reported by Guillaume et al. [25]. There was a low rate of Nipah virus extracellular RNA release (~0.07 log PFU/μL per hour) over a period of 56 hours. This gradual RNA release that peaked only at 64 hours PI, suggests that Nipah virus particles were not immediately released into the cell culture medium, perhaps not until most cells could no longer sustain further infection. This observation is consistent to that which had been described for measles and canine distemper virus, viruses known to spread through cell-to-cell contact [32-34] and similar to these viruses [34-37], results from the present study suggest that only limited budding of virus particles and low production of infectious virus occurred during the early stages of infection. The formation of extensive multinucleated giant cells that increased in number with time and the lack of cell lysis throughout the 72 hours period of Nipah virus infection further support the possibility that Nipah virus infection is spread through cell-to-cell contact mechanism.

## Conclusion

Nipah virus replication kinetics in Vero cells was established using the SYBR^® ^Green I-based qRT-PCR assay. A short viral RNA doubling time was observed but the rate of extracellular virus RNA and infectious virus release from the infected cells were low. These suggest that Nipah virus replicates well in susceptible cells but the infection is insidious as the virus is spread slowly through the cell-to-cell spread mechanism.

## Materials and methods

### Nipah virus inoculum and virus titration

Vero cells used for Nipah virus isolation were cultured in Eagle's minimum essential medium (EMEM; Flowlab, Australia) supplemented with 2% fetal calf serum (FCS, BioWhittaker, USA). Cells were incubated at 37°C in 5% CO_2 _and infected with the pig Nipah virus strain NV/MY/99/VRI-2794. The infected cells were examined for cytopathic effects (CPE). Following manifestation of ~90% CPE, the supernatant was harvested and sedimented at 1000 × g to remove all residual cells. The supernatant was then stored at -80°C. The supernatant was later titrated for virus infectivity using virus plaque assay. Briefly, a tenfold serial dilution of the virus stock was prepared and 250 μL of each virus dilution was added in duplicates into 24-well plate containing 1 × 10^6 ^cells/well. The virus-cells mixture was incubated at 37°C for one hour. Following that, cells were washed twice with EMEM. Then, 500 μL of 0.8% agarose in EMEM supplemented with 2% FCS was overlaid on top of the cell monolayer and the plate was incubated at 37°C. On day two PI, the virus-cells mixture were fixed with 4% paraformaldehye and stained with naphthalene black. Virus infectivity titer was estimated by determining the virus dilution and the number of plaques formed.

### Preparation of RNA for quantitative real-time amplification

Nipah virus RNA was extracted from Nipah virus inoculum following determination of the virus infectivity titer. RNA was extracted using the TRI Reagent^® ^LS (Molecular Research Centre, Inc., USA) according to the manufacturer's protocol. The RNA pellet was dissolved in nuclease-free water and a tenfold serial dilutions of the RNA was made to reflect the calculated PFU of 0.1 to 1 × 10^6 ^for establishing the qRT-PCR assay standard plot. In addition, Nipah virus Armored RNA^® ^(Ambion, USA) containing ~5 × 10^5 ^Nipah virus RNA copies per μL (Lot #10233) was used for the estimation of Nipah virus RNA copy number. The RNA was prepared according to the manufacturer's instructions to generate 0.5 to 5 × 10^5 ^copies of the Nipah virus RNA.

### RT-PCR for amplification of Nipah virus N gene sequence

Initial amplification of Nipah virus sequences was accomplished using the conventional RT-PCR performed in a PTC-200 thermal cycler (Bio-Rad Laboratories, Inc., USA). Primer pairs NIP-NF3 (5' GGC TAG AGA GGC AAA ATT TGC TGC 3') and NIP-NR1 (5' ACC GGA TGT GCT CAC AGA ACT G 3'), designed from the conserved region within the N gene were used. The reaction mixture consisted of 1× Access RT-PCR buffer (Promega, USA), 0.5 μL MMV, 0.5 μL AMV, 1 mM of dNTPs, 1.5 mM of MgSO_4_, 0.6 pmol/μL of each primer, and 1 μL template RNA. Amplification was performed in a 25 μL reaction mix using the following program: 42°C of cDNA synthesis for 1 hour, 95°C for 15 min, 30 cycles of 1 min at 95°C, 1 min at 55°C, and 1 min at 72°C and followed by final extension of 72°C for 5 min.

The SYBR^® ^Green I-based qRT-PCR was performed using the same set of oligonucleotide primers as above, in a 20 μL mix at 50°C for 30 min, 95°C for 15 min, and 45 cycles of 15 s at 95°C and 1 min at 60°C. The reaction mixture consisted of 1× QuantiTect SYBR^® ^Green RT-PCR Master Mix (Qiagen, Germany), 0.5 μL QuantiTect RT Mix, 0.6 pmol/μL of each primer, and 1 μL template RNA. The amplification was performed in DNA Engine Opticon^® ^System (Bio-Rad Laboratories, Inc., USA). Fluorescent measurements were recorded after each cycling step and at the end of the amplification cycle data were analyzed using the OpticonMONITOR™ 2 analysis tool. A threshold cycle (Ct) value for every sample was determined and compared to that of the standard. The standard plot was established in parallel for each experiment using known amount of Nipah virus Armored RNA^®^. Standard curves for the RT-PCR was accepted when the coefficients of correlation, r^2 ^were > 0.90. All amplification standards, controls, and samples were performed in duplicates and repeated at least twice. In addition, melting curve analysis was performed routinely at the end of each amplification assays to verify the amplicon by its specific T_m_. The melting curve analysis consisted of 35 cycles of incubation during which the temperature was increased from 60°C to 95°C at a rate of 0.2°C/30 s/cycle with continuous reading of fluorescence.

### Kinetics of Nipah virus RNA synthesis in Vero cells

Adherent Vero cells (2.5 × 10^5 ^cells/well) cultured in 24-well plate were infected with Nipah virus to give an estimated MOI of 0.2 per cell. Cells were incubated with the virus for one hour at 37°C, following which the virus suspension was removed and the cells were rinsed twice with EMEM. Subsequently, EMEM supplemented with 2% FCS was added and the cells were incubated at 37°C. At selected intervals PI (every eight hours, from zero to 80 hours) the cell culture supernatant consisting of the extracellular virus was removed, centrifuged at 1000 × g and RNA was extracted using TRI Reagent^® ^LS (Molecular Research Centre, Inc., USA). The remaining cell monolayer was rinsed twice with serum free EMEM medium and total intracellular RNA was extracted using the TRI Reagent^® ^(Molecular Research Centre, Inc., USA). The efficiency of RNA extraction was consistent between all extractions at ~72.3% (± 1.5%). All the extracted RNA was stored at -70°C until needed.

## Competing interests

The author(s) declare that they have no competing interests.

## Authors' contributions

The corresponding author, Sazaly AbuBakar is the principal investigator of the study, was involved in the design, supervision, data analyses and writing of the report. Li-Yen Chang performed all the laboratory experiments, analyses of data and writing of the report. A.R. Mohd Ali contributed in the virological investigations. Sharifah Syed Hassan was involved in the virological investigations and supervision for the usage of the BSL 3 facility.
